# A prospective multicenter evaluation of direct molecular detection of blood stream infection from a clinical perspective

**DOI:** 10.1186/s12879-016-1646-4

**Published:** 2016-06-30

**Authors:** A. E. Nieman, P. H. M. Savelkoul, A. Beishuizen, B. Henrich, B. Lamik, C. R. MacKenzie, D. Kindgen-Milles, A. Helmers, C. Diaz, S. G. Sakka, R. P. Schade

**Affiliations:** Department of Medical Microbiology and Infection control, VU University Medical Center, Amsterdam, The Netherlands; Department of Medical Microbiology, Maastricht University Medical Center, Maastricht, The Netherlands; Department of Intensive Care Medicine, VU University Medical Center, Amsterdam, The Netherlands; Department of Intensive Care, Medisch Spectrum Twente, Enschede, The Netherlands; Institute of Medical Microbiology and Hospital Hygiene, University Clinic of Heinrich-Heine University Düsseldorf, Düsseldorf, Germany; Department of Anesthesiology, University Clinic of Heinrich-Heine University Düsseldorf, Düsseldorf, Germany; Department of Microbiology, Merheim Zentrallabor, MVZ synlab Leverkusen GmbH, Leverkusen, Germany; Department of Anesthesiology and Intensive Care Medicine, Medical Center Cologne-Merheim, University Witten/Herdecke, Cologne, Germany; Laboratory for Medical Microbiology, Immunology and Infection Control, Elisabeth-TweeSteden Hospital, Tilburg, The Netherlands

**Keywords:** SepsiTest™, PCR, Blood stream infection, Sepsis, Bacteremia, Molecular, 16S

## Abstract

**Background:**

Rapid diagnosis and appropriate antimicrobial therapy are of major importance to decrease morbidity and mortality in patients with blood stream infections (BSI). Blood culture, the current gold standard for detecting bacteria in blood, requires at least 24–48 hours and has limited sensitivity if obtained during antibiotic treatment of the patient. The aim of this prospective multicenter study was to clinically evaluate the application of a commercial universal 16S/18S rDNA PCR, SepsiTest™ (PCR-ST), directly on whole blood.

**Methods:**

In total 236 samples from 166 patients with suspected sepsis were included in the study. PCR-ST results were compared to blood culture, the current gold standard for detecting BSI. Because blood cultures can give false-negative results, we performed an additional analysis to interpret the likelihood of bloodstream infection by using an evaluation based on clinical diagnosis, other diagnostic tests and laboratory parameters.

**Results:**

Clinical interpretation of results defined the detected organism to be contaminants in 22 of 43 positive blood cultures (51.2 %) and 21 of 47 positive PCR-ST results (44.7 %). Excluding these contaminants resulted in an overall sensitivity and specificity of the PCR-ST of 66.7 and 94.4 % respectively. Of the 36 clinically relevant samples, 11 BSI were detected with both techniques, 15 BSI were detected with PCR-ST only and 10 with blood culture only. Therefore, in this study, SepsiTest™ detected an additional 71 % BSI compared to blood culture alone.

**Conclusions:**

More clinically relevant BSI were diagnosed by molecular detection, which might influence patient treatment. An improved SepsiTest™ assay suited for routine use can have additional value to blood culture in diagnosing bacteremia in septic patients.

## Background

Blood stream infection is a serious clinical condition with high morbidity and mortality rates. Sepsis, severe sepsis and septic shock are associated with high mortality, ranging from 20 to 60 % depending on severity and underlying disease [1–4]. Sepsis is defined as a systemic inflammatory response syndrome (SIRS) in addition to documented or presumed infection. Severe sepsis is sepsis associated with organ dysfunction, hypoperfusion, or hypotension. Septic shock is the persistence of hypotension and perfusion abnormalities despite adequate resuscitation therapy [5]. Bacteremia, the presence of bacteria within the bloodstream, which leads to SIRS or sepsis, is associated with high morbidity and mortality. Rapid diagnosis of BSI and early initiation of appropriate antimicrobial treatment significantly decreases morbidity and mortality. Appropriate therapy in septic patients within the first hour of documented hypotension was associated with a survival rate of 80 %, with an average decrease of 7,6 % in survival every hour, up to 6 h, that antibiotics were delayed [6]. Empiric antimicrobial therapy is correct in approximately 85–90 % of blood stream infections [7–10]. As soon as microbiological results are available, inappropriate antibiotics can be adjusted.

Currently, automated blood culture systems are the standard technique to diagnose BSI, however, blood cultures have several disadvantages. First, the minimum time to detection and identification of the causative organism is 24–48 h. Secondly, the sensitivity is approximately 70 % for critically ill patients and even lower for fastidious microorganisms [11]. Thirdly, prior administration of antibiotics significantly reduces the sensitivity of blood culture, sometimes even up to 100 % [12–15].

These disadvantages can potentially be overcome by molecular techniques performed directly on blood without culture. Polymerase chain reaction (PCR) targeting microbial DNA performed directly on blood is a rapid technique with a processing time of only a few hours. PCR detection of bacterial or fungal DNA is not dependent on the presence of replicating organisms and can be detected from intact dead or living bacteria. The major technological drawback to apply PCR assays for diagnosing blood stream infection is inhibition of the PCR amplification by relatively large amounts of proteins and human DNA in whole blood samples that currently limits the sample volume to approximately 200 μl of whole blood [14]. Human DNA in leukocytes and proteins, like immunoglobulin G, heme in erythrocytes and lactoferrin in leukocytes all interfere with the process by binding DNA polymerase or binding the nucleic acids [16–21]. However, optimization of DNA isolation and new methods that selectively purify microbial DNA to circumvent inhibition by human DNA have been developed. In this way higher volumes of blood can be processed which theoretically reduces sampling error and increases sensitivity. SepsiTest™ is not the only technique in this field, several other commercial tests have been developed that use PCR or time-of flight mass spectrometry for fast identification of pathogens, i.e. SeptiFast, MALDI-TOF on blood culture and more recently PCR ESI/MS (IRIDICA). The SeptiFast assay is a multiplex PCR performed directly on whole blood. Sensitivity compared to blood culture was 12–67 % depending on the microorganism, with a specificity of 81–98 % [22, 23] and the clinical sensitivity of the SeptiFast assay, excluding contaminants, was 62–70 % [24]. A recent randomized trial showed a significant reduction in the time required from sampling to providing pathogen identification to the clinician, with a mean duration of 15.9 h for PCR compared to 38.1 h for blood culture [25]. A drawback of this technique is the limited number of bacterial and fungal pathogens, 25 in total, in this multiplex PCR, and the low sensitivity. The next technique, MALDI-TOF in combination with the commercial Sepsityper kit allows identification on positive blood cultures with a reliable detection of 76 % of Gram-positive microorganisms and 90 % of Gram-negative microorganisms [26]. Of course the major drawback of this method is the necessity to culture first, which takes approximately 1–2 days and delays the time-to-result compared to direct tests on blood. More recently IRIDICA has been developed, a technique that combines multiplex PCR directly on whole blood with electrospray ionization mass spectrometry (PCR ESI/MS). Sensitivity and specificity compared to blood culture were 83 and 78–94 % and sensitivity and specificity of the test compared with the clinical infection criterion were 91 and 87 % [27, 28]. This seems a promising technique, however publications so far are limited.

We here present a prospective multicenter study that investigates the SepsiTest™ assay (Molzym, Bremen, Germany) in 1 ml of whole blood from patients on intensive care units with clinically suspected sepsis. The SepsiTest™ assay selectively degrades human DNA, before isolation of the microbial DNA. SepsiTest™ can provide a positive or negative result within 4 h and needs additional sequencing to identify the microorganism, which takes another 2–3 h if sequencing is available in the laboratory. Clinical relevance and routine applicability of this approach was evaluated by comparing the PCR results to blood culture, C-reactive protein, other microbiological investigations and patient data. This study shows that molecular detection of BSI has a potential benefit for patients suffering from sepsis however, several improvements of the technique are still required.

## Methods

This study was conducted as a multicenter trial with patients suspected of sepsis. From each patient routine blood cultures and EDTA blood for PCR with the SepsiTest™ assay were taken. The study was performed in three participating centers: Medical Center Cologne-Merheim, University Witten/Herdecke in Cologne, Germany; Heinrich Heine University Hospital in Düsseldorf, Germany and VU University Medical Center in Amsterdam, The Netherlands. The study was approved by the Institutional Ethical Board of each center. Blood sampling was incorporated in routine patient care, without additional written informed consent. Blood samples for PCR-ST were collected; DNA extraction, PCR and sequencing were performed retrospectively. As this was an observational study, results of PCR-ST were not shared with the clinicians. Treatment of the patient was based on cultures and clinical signs and symptoms.

### Patient selection

In total 166 patients were included in the three centers. The collection period was 5 months for each center, in the time period from November 2010 to September 2012. Inclusion criteria were patients ≥18 years old, admitted to the intensive care unit with systemic inflammatory response syndrome (SIRS) or suspected sepsis. If the treating physician had a clinical suspicion of SIRS or sepsis, blood was taken for culture and PCR-ST.

### Sample and data collection

Samples for blood culture and PCR-ST were collected from the same blood vessel puncture according to the hospital’s standardized aseptic procedures. For blood culture 10 ml of blood was drawn into an aerobic and anaerobic blood culture bottle each, and inoculated in BACTEC™ FX (Beckton Dickinson) or BacT/ALERT® (bioMérieux) depending on the participating center. Blood cultures were incubated for a maximum of 5 days. For PCR-ST, an additional volume of 4 ml of EDTA blood was collected. Identification of the organism in positive blood culture was performed with conventional methods as customary in each laboratory. All laboratories were certified according to the national quality standard for clinical microbiological laboratories.

Other samples were taken according to the suspected cause of infection, e.g. sputum, wound swabs, pus or urine. C-reactive protein and leucocyte count were recorded on the day of blood sample collection. Primary and secondary diagnosis, antibiotics used in the past 48 h and hospital inpatient mortality were registered.

### DNA isolation with SepsiTest™

Fresh EDTA blood was divided into two aliquots of 1 ml and processed according to the SepsiTest™ manufacturer’s manual. In short, a chaotropic buffer was first added to the blood samples that selectively lysed the blood cells. Then, the released nucleic acids were degraded by adding MolDNase. After enrichment by centrifugation, the sediment was treated with BugLysis and ß-mercaptoethanol to lyse cell walls of potentially present bacteria and yeasts. Proteinase K and a final chaotropic buffer was added for extra cell lysis and denaturation of proteins. The pathogen DNA was isolated by a bind-wash-elute procedure and eluated in a final volume of 100 μl. All materials used during these steps were sterile and nucleic acid-free to avoid laboratory and handling contamination. Sterile and nucleic acid free buffers and tubes were provided in the SepsiTest™ kit. Sterile pipette tips were used. Handling of samples was performed in a laminar flow cabinet that was disinfected and treated with UV light after each use.

### PCR and amplicon detection

The protocol for PCR-ST was performed according to the manufacturer’s manual. Mastermixes for universal PCR, internal control, and negative and positive controls were available in the kit. Five μl of eluate (~50 μl EDTA blood) of each duplicate sample were tested, with internal inhibition tested separately. All PCR-STs were performed on Piko Thermal Cyclers (Finnzymes). Amplicons were detected with electrophoresis on 2 % agarose gel. A sample was considered positive if at least one of the duplicates was positive with a blank negative control.

### Sequencing

Positive amplicons were purified with QIAquick PCR Purification Kit (Qiagen) and sent to GATC Biotech (Germany) for sequencing with primers provided in the kit. Acquired sequences were identified with SepsiTest™ BLAST, or with NCBI BLAST when results were not available in SepsiTest™ BLAST. The organism was identified at a genus level if the sequence homology was ≥97 and <99 % and at a species level if ≥99 % according to the manufacturer’s manual. For samples with weak bands on agarose gel and insufficient quality of sequencing results, the PCR, amplicon detection and sequencing were repeated. If sequencing results still had insufficient quality, the sample was considered negative.

### Definitions

Positive samples were divided into 4 categories: true, probable, possible and questionable bacteremia. Identical bacteria detected both in blood culture and PCR-ST was considered as true bacteremia. For discordant results an interpretation of the likelihood of bacteremia was made according to the following definitions: bacteremia was considered probable if the pathogen was also cultured in other specimens of the same patient and consistent with the clinical diagnosis; possible if the pathogen was not confirmed in other cultures, but considered to be consistent with the clinical diagnosis. The result was interpreted as questionable bacteremia if the microorganism was not confirmed by other microbiological results and was not considered a likely cause of sepsis. In this case we defined the microorganism as a contaminant.

Results of other specimens, i.e. urine culture, wound swabs etc., were taken into account when sampled within 48 h before or after the positive blood culture or positive PCR-ST.

A polymicrobial result, i.e. a sample which yielded more than one microorganism in the same sample, was considered as true, probable or possible bacteremia, when one of the detected microorganisms met the definitions for true, probable or possible.

### Statistical analysis

Sensitivity and specificity of PCR-ST by using blood culture as gold standard were calculated both on sample and patient level. Statistics were calculated based on paired samples, i.e. blood culture with corresponding PCR-ST sample. Subsequently, to investigate the potential additional value of PCR-ST in diagnosing BSI, the sensitivity and specificity of the PCR-ST were also calculated including clinical interpretation of results. Based on the above definitions, positive samples were divided into: true, probable, possible and questionable BSI. True, probable and possible BSI were regarded as positive, and questionable as negative for the calculation of an adjusted sensitivity and specificity.

## Results

In this study, 166 patients were included with a total of 236 blood samples. The mean age of the patients was 65 years, with a range from 26 to 92 years. Sixty-three women were included (38 %). From all blood samples 43 of the 236 were positive by blood culture (18.2 %) and 47 were positive by PCR-ST (19.9 %). Samples matched in 72 % of cases. After excluding contaminants 91 % of the samples matched.

Sensitivity and specificity of the SepsiTest™ assay compared to blood culture was 25.6 and 82.9 % respectively, with negative and positive predictive values of 84.7 and 23.4 % (sample level, Table 1). When analyzed for the 166 patients, sensitivity and specificity were 28.9 and 79.7 %, with a negative and positive predictive value of 81.0 and 27.5 % (patient level).

**Table 1:** Results of BC and PCR-ST without clinical interpretation

	BC positive	BC negative	Total
PCR-ST positive	11 + 3^a^	33	47
PCR-ST negative	29	160	189
Total	43	193	236

*Abbreviations*: *BC* blood culture, *PCR-ST* polymerase chain reaction with SepsiTest™

^a^Three samples were positive in both BC and PCR-ST, however the microorganisms detected were different. For calculating sensitivity and specificity of PCR-ST compared to BC only concordant results were included

The majority of detected microorganisms were staphylococci in both blood culture and PCR-ST (Table 2). Coagulase negative staphylococci (CoNS) were found in blood cultures in 30 of the 43 positive cultures (69.8 %). *Staphylococcus aureus* was found in 2 blood cultures. For PCR-ST, identification to species level was not always possible. CoNS were detected by PCR-ST in 13 of 47 positive samples (27.7 %) whereas *S. aureus* was detected in 6 cases (12.8 %). *Staphylococcus* spp. not further identified, were detected in an additional 9 samples (19.1 %). It was not possible to distinguish between coagulase negative staphylococci or *Staphylococcus aureus* for these 9 samples, due to insufficient quality of the sequencing fragment at the site of distinction. In total, 32 staphylococci (74.4 %) were detected with blood culture compared to 28 (59.6 %) with PCR-ST. Gram-negative rods were found in two samples with blood culture (4.7 %) and in six samples with PCR-ST (12.8 %). Candida was found in only one sample with each technique, however in different samples.

**Table 2:** Microorganisms detected in sepsis patients with PCR-ST alone, blood culture alone or detected with both methods

True, probable and possible bacteremia			
	PCR-ST	BC	both
Monomicrobial			
*Staphylococcus aureus*	3	1	1
Coagulase negative *Staphylococci* (CoNS)	2	3	5^a^
*Staphylococcus* spp.	1	0	1
*Enterococcus* spp.	1	3	3^b^
*Streptococcus pneumoniae*	1	0	0
*Escherichia coli*	1	1	0
*Klebsiella oxytoca*	0	0	1
*Candida albicans*	1	1	0
Polymicrobial^c^	5	1	0
Questionable bacteremia			
	PCR-ST	BC	
Monomicrobial			
Coagulase negative *Staphylococci*	5	20	
*Staphylococcus* spp.	3	0	
*Propionibacterium* spp.	0	2	
*Streptococcus* spp.	4	0	
*Enterococcus* spp.	1	0	
*Lactococcus lactis*	1	0	
*Rickettsia symbiont*	1	0	
*Corynebacterium* spp.	1	0	
Polymicrobial^c^	5	0	
	PCR-ST	BC	both
Total	36	32	11

*Abbreviations*: *PCR-ST* polymerase chain reaction with SepsiTest™, *BC* blood culture, *spp.* species

^a^one sample contained CoNS in blood culture and CoNS + Corynebacterium in PCR-ST

^b^one sample contained *Enterococcus faecalis* in blood culture and *Enterococcus* + *Streptococcus* spp in PCR-ST

^c^for microorganisms detected in polymicrobial samples, see Table 3

In total 236 samples were analyzed of which 79 were positive in one or both methods. Results are presented by likelihood of bacteremia, true/probable/possible versus questionable bacteremia

Polymicrobial samples, i.e. samples with more than one microorganism, were detected in only one blood culture (2.3 %), but were common in the PCR-ST results (12 samples, 25.5 %) (Table 3). It is to be noted that 9 out of 12 polymicrobial PCR-ST results were found in one center (75 %).

**Table 3:** Microorganisms detected in polymicrobial samples with PCR-ST and blood culture

True, probable and possible bacteremia		
	PCR-ST	BC
Polymicrobial		
*Enterococcus* spp, Coagulase negative *Staphylococci*	0	1
*Staphylococcus aureus*, *Lactobacillus* spp.	1	0
*Escherichia coli*, *Streptococcus mitis* group	1	0
*Pseudomonas aeruginosa*, *Acinetobacter lwoffii*, *Staphylococcus warneri*, *Streptococcus equi*	1	0
*Escherichia coli*, *Edwardsiella ictaluri*, *Enterococcus faecium*, *Staphylococcus aureus*	1	0
*Streptococcus agalactiae*, *Enterococcus* spp, *Propionibacterium* acnes, *Staphylococcus* spp.	1	0
Questionable bacteremia		
	PCR-ST	BC
Polymicrobial		
*Lactobacillus* spp., *Enterococcus mundtii*	1	0
*Haemophilus parainfluenzae*, *Haemophilus ducreyi*, *Neisseria wadsworthii*, *Lactococcus lactis*	1	0
*Staphylocccus* spp., *Streptococcus* spp	1	0
*Lactobacillus* spp., *Staphylocccus* spp., *Streptococcus* spp.	1	0
*Abiotropha paraadiacens*/*Granulicatella adiacens*, *Staphylococcus* spp., *Streptococcus* spp.	1	0
	PCR-ST	BC
Total	10^a^	1

*Abbreviations*: *PCR-ST* polymerase chain reaction with SepsiTest™, *BC* blood culture, *spp.* species

^a^Two additional polymicrobial PCR-ST results are listed in Table 2: these samples contained two microorganisms in the PCR-ST and one concordant microorganism in the corresponding BC

A polymicrobial sample, a sample that yielded more than one microorganism, was considered as true, probable or possible bacteremia, when one of the detected microorganisms met the definitions for true, probable or possible, regardless of the meaning of the other microorganisms

Evaluation of PCR-ST results to blood culture as gold standard may result in lower sensitivity when clinically contaminated blood cultures are defined as positive. On the other hand, specificity may be hampered by false negative blood cultures due to antibiotic therapy or bacteria that are difficult to culture. Based on the combination of lab results and clinical interpretation of the microorganism with respect to the patient’s disease, 22 of 43 positive blood cultures of in total 236 samples were considered to be contaminants. This is 51,2 % of positive blood cultures and 9,3 % of all blood cultures. For PCR-ST 21 of 47 positive results were considered to be contaminants (44,7 %), i.e. 8,9 % of all samples. For example, a septic patient with the clinical signs of a thrombophlebitis of the lower arm and a *Staphylococcus aureus* detected in both blood culture and PCR-ST, was considered to have a true bacteremia. A patient with peritonitis and a negative blood culture, however *Enterococcus* spp. detected with PCR-ST that had also *Enterococcus faecium* in peritoneal cultures was considered to have probable bacteremia. On the other hand, a *Corynebacterium* detected with PCR-ST that was not detected in any other culture and is known to be a common contaminant, was considered as questionable bacteremia. After clinical interpretation of all positive results (Table 4), the adjusted sensitivity and specificity on sample level was 66.7 and 94.4 %. The negative and positive predictive value was 96.7 and 53.8 % respectively. In total 36 samples were considered clinically relevant as detected by PCR-ST, blood culture or both. Of these 36 samples, 11 microorganisms were detected by both methods, 15 were detected by PCR-ST only and 10 by blood culture only.

**Table 4:** Results of BC and PCR-ST after excluding contaminants

	BC positive	BC negative	Total
PCR-ST positive	14^a^	12	26
PCR-ST negative	7	203	210
Total	21	215	236

*Abbreviations*: *BC* blood culture, *PCR-ST* polymerase chain reaction with SepsiTest™

^a^Three samples were positive in both BC and PCR-ST, however the microorganisms detected were different. Since both microorganisms were considered clinically relevant these samples were included as possible/probable blood stream infections in the adjusted sensitivity

Of 28 patients with clinically relevant positive blood samples, 13 (47 %) had a respiratory or an intra-abdominal cause of sepsis (Table 5). From these patients 22/28 (78.6 %) had received antibiotics in the 48 h prior to testing. Of discordant results, i.e. a positive result in either blood culture or PCR-ST alone, 8 patients with previous antibiotics had a PCR-ST positive result only, compared to 6 patients with positive blood cultures only.

**Table 5:** Focus of infection in patients with positive blood culture and/or PCR results considered true, probable or possible

Focus of infection	Number of patients
Intra-abdominal	8
Respiratory	5
Cardiac	3
Central nervous system	3
Skin	2
Postoperative fever	2
Trauma	2
Intravascular catheter	1
Multiorgan failure	1
Bone	1
Total	28

This study clearly indicates that clinical interpretation of PCR-ST results is important, as is true for blood culture. The additional value of PCR-ST in diagnosing BSI is substantial; an extra 15 bacteremias confirming the clinical perspective were detected compared to 21 with blood culture alone (71 %). These results may influence antimicrobial therapy in terms of type, dose and duration.

## Discussion

A rapid diagnosis of BSI is of major importance for patient outcome. A direct PCR on whole blood, instead of blood culture, should theoretically reduce the time to diagnosis and implementation of adequate antimicrobial therapy.

In this multicenter study we have evaluated the performance of a universal PCR assay in patients suspected of sepsis. Sensitivity and specificity were based on comparing PCR-ST with blood culture, on both sample and patient level. To determine the actual clinical relevance of these PCR-ST results, all positive samples were also analyzed after a clinical interpretation of the microorganism detected in respect to other diagnostic tests, and the patient’s disease. Despite the fact that the total numbers of relevant microorganisms were small, with 21 relevant blood cultures and 26 relevant PCR-ST results in a total of 236 samples, the performance of the SepsiTest™ within a clinical context was verified.

Sensitivity of PCR-ST compared to blood culture, without clinical interpretation of results, was only 33 % with a specificity of 83 % (sample level). A previous study showed similar specificity (85.8 %), but much higher sensitivity (87 %) of the SepsiTest™ assay compared to blood culture [29]. The same test and a comparable patient population were evaluated but we found a higher number of blood cultures with coagulase negative staphylococci that were considered to be a contamination (47 % of positive BC). In routine application, clinical interpretation of results is crucial for clinical use of both blood culture as well as PCR-ST. In 9 of 47 positive PCR-ST results *Staphylococcus* spp. were found. Due to insufficient quality of the sequencing fragment at the site of distinction it was not possible to differentiate between *Staphylococcus aureus* and coagulase negative staphylococci. This is a shortcoming of the test, since coagulase negative staphylococci are often contaminants while *Staphylococcus aureus* is a clinically relevant pathogen. More than 40 % of positive results in both blood culture and PCR-ST were considered contaminants, based on clinical diagnosis, laboratory parameters and other microbiological tests performed in the same individual. In the majority of these results coagulase negative staphylococci were found in blood culture and staphylococci and streptococci were found in PCR-ST. A recent study of 1334 patients with positive blood cultures also showed significant numbers of contaminants. In this study 55,8 % of blood cultures were considered bacterial contamination, compared to 44,2 % true blood stream infections [15]. After clinical interpretation of results, the adjusted sensitivity and specificity were 66.7 and 94.4 % respectively. For clinical interpretation we made some assumptions as described in the methods. Questionable bacteremia as defined in this study may actually be true bacteremia and possible bacteremia may be actually contaminants. This is a drawback of the calculated adjusted sensitivity and specificity. However, in daily practice it is very common to make a clinical evaluation of the microorganism found in the blood culture, especially for coagulase negative staphylococci and certain streptococci. For PCR-ST a clinical interpretation is also important. Interpretation errors may limit these calculations.

In studies evaluating PCR directly on whole blood, other than SepsiTest™, low sensitivities were also found compared to blood culture results. For the SeptiFast assay, Josefson and Grif showed a sensitivity on sample level of 12–67 and 63 % respectively [22, 23]. Clinical sensitivity, after excluding contaminants, was 62–70 % (Bravo et al.) [24]. In these studies a multiplex PCR with a select number of microorganisms was used to detect bacteremia. Bacteria that were not included in the multiplex PCR were logically not detectable with this approach. However, this explained only in part the low sensitivity since the majority of microorganisms missed were included in the PCR panel but were only detected by blood culture [22–24, 30].

An explanation for the low sensitivity in most studies as well as in our study is possibly the large difference in input volume used in blood culture compared to PCR. For one blood culture bottle, 8–10 ml of blood was used. For PCR, an amount equal to 50 μl of whole blood was tested. This means that the amount of blood tested in one blood culture bottle is around 200 times more than in one PCR reaction. The chance of sampling error in PCR is therefore much higher, especially when low amounts of bacteria are present in the blood of the patient. This can be improved by using 50 μl of eluate and 10 μl of DNA in the PCR although the amount of blood tested in a blood culture bottle will still be 50 times more. If higher input volumes for PCR can be used, sensitivity of PCR on blood will increase [16–21].

Specificity was more in line with blood culture, however it is debatable whether blood culture is a reliable gold standard. In our samples we found many staphylococci and considered more than 50 % of positive blood cultures (mostly CoNS) clinically irrelevant. On the other hand, blood cultures may be false negative. The sensitivity of blood culture increases if more blood cultures are obtained, from approximately 65–73 % for 1 set of blood culture bottles (20 ml), 80–90 % for two sets and 95–99 % for 3 sets of blood cultures. As a consequence one negative blood culture cannot exclude BSI [31, 32]. Therefore, the presence of microorganisms detected by PCR may reflect actual disease. Moreover, the microorganisms can truly be temporarily present in blood, as is known for short bacteremias after dental procedures [33–35] and surgical procedures [36, 37]. Mostly, these bacteremias do not cause a systemic inflammatory response syndrome and therefore no BSI. These are true bacteremias, but not clinically relevant. Of course, in a universal PCR technique the chance of contamination of the sample in the laboratory is also substantial. Clean handling of samples throughout the entire procedure from withdrawal to PCR is of utmost importance. Obviously, for both PCR-ST and blood culture, the detected microorganisms need to be evaluated in the clinical context to decide whether the result is more likely to be clinically relevant or contamination.

Theoretically, the advantage of blood culture is that one viable bacterium is sufficient for a positive result. High volumes of blood can be tested at once, and the possibility of testing antibiotic susceptibility makes blood culture a valuable technique. PCR on the other hand is useful for microorganisms that are difficult or impossible to culture, after the initiation of antibiotic therapy and can be performed directly on whole blood, thus eliminating the incubation time, which is important for early adequate antimicrobial therapy.

The SepsiTest™ assay is a complex method with many buffers, enzymes and reagents to be added and several centrifugation steps before the eluate is obtained. Hands-on-time and the possibility of laboratory contamination can be reduced by automation of this procedure and replacing gel electrophoresis by melting curve analysis to detect positive samples. The SepsiTest™ assay is now designed as a positive or negative result with subsequent sequencing to identify the bacteria. DNA extraction and PCR-ST can be performed within 4 h and produces a negative or positive result. Only a positive result needs to be sequenced for identification of the microorganism. This adds 2 to 3 h to the time-to-results, provided that sequencing is available in the laboratory. In our study, sequencing was outsourced which increased the turnaround time. In routine practice however this could be performed within 6–7 h until complete identification. The test can be used without sequencing, with only a positive or negative result. However, owing to the substantial chance of contamination, a clinical interpretation of results is necessary and therefore the identification of the microorganism. With the current setup of this method, the theoretical advantage of a fast PCR directly on whole blood is reduced by the complex handling procedure and the need for sequencing to identify the microorganism.

## Conclusions

This study in 166 patients with sepsis indicates that direct molecular detection of bacteria is of added value to blood culture in detecting blood stream infections. An additional 71 % of BSI were detected by the SepsiTest™ assay (PCR-ST) compared to blood culture alone. Furthermore, after antibiotic therapy, slightly more BSI were detected by PCR-ST than by blood culture. Overall, PCR-ST results may influence the administration of adequate antimicrobial therapy and diminish patient’s morbidity and mortality. Although the SepsiTest™ PCR directly on blood is a promising technique, the input volume of blood should be increased to lower sampling error, and a faster procedure to identify the microorganism is of importance. The current universal microbial PCR is not a substitute for blood culture but may be of additional clinical value in diagnosing BSI. In general, from a clinical perspective a rapid molecular whole blood test is of added value for patient treatment.

## Abbreviations

BC, blood culture; BSI, blood stream infection (s); CoNS, coagulase negative staphylococci; ESI/MS, electrospray ionization mass spectrometry; MALDI-TOF, matrix assisted laser desorption/ionization time-of-flight (mass spectrometry); PCR, polymerase chain reaction; PCR-ST, universal PCR with SepsiTest™; SIRS, systemic inflammatory response syndrome.
